# Recent Patents of Interest to Dentists

**Published:** 1907-02

**Authors:** 


### RECENT PATENTS OF INTEREST TO DENTISTS.

839920. Magnesia-cement composition, Wm. L. Dudley, Nashville, Tenn.
840348. Specialists' chair, Charles N. Leonard, Indianapolis, Ind.
840738. Dentifrice, Frank P. Barnard, Worcester, Mass.
840921. Dental swage, Joseph W. Dickey, St. Louis, Mo.
841006. Apparatus for the manufacture of reducing amalgam, Herbert P. Ewell, Rochester, Mich.
38408. Design, dental cabinet, Harry C. Gowran, Two Rivers, Wis.
38409. Design, dental cabinet, Harry C. Gowran, Two Rivers, Wis.

Copies of above patents may be obtained for fifteen cents each by addressing John A. Saul, Solicitor of Patents, Fendall Building, Washington, D. C.

